# Supplementary material for: High-speed imaging of light-induced photoreceptor microsaccades in compound eyes
Source: Commun Biol. 2022 Mar 3;5:203. doi: 10.1038/s42003-022-03142-0 (PMC8894348; doi:10.1038/s42003-022-03142-0)
Supplement: Supplementary file 2 — Supplementary Information [file 42003_2022_3142_MOESM2_ESM.pdf]

High-speed imaging of light-induced photoreceptor microsaccades in compound eyes

Joni Kemppainen[1], Neveen Mansour[1], Jouni Takalo[1] & Mikko Juusola[1,2*]

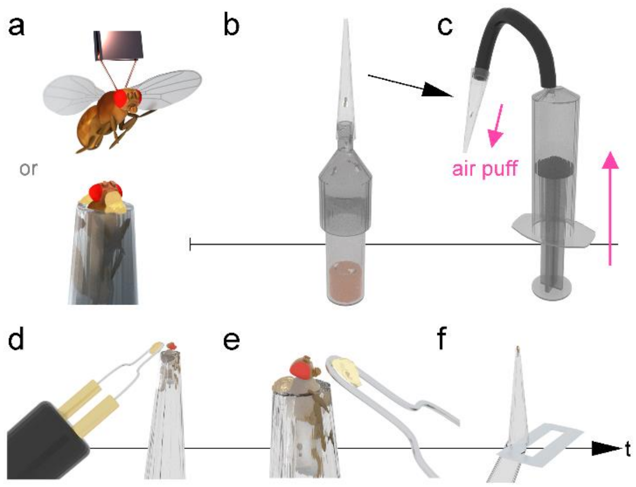

Supplementary Fig.1 | The pipette tip preparation technique. **a**, Flies can be either tethered on a small copper hook with UV-cured glue or immobilised on a plastic pipette tip with beeswax. **b**, With the help of a funnel piece, a fly was captured in a 1,000 µl plastic pipette tip. **c**, Air puffs generated by a hand-held syringe pushed the fly towards the pipette tip's small end. **d**, The fly was immobilised from its dorsal thorax. **e**, the head was secured near the proboscis using melted beeswax. **f**, The pipette excess was cut off with a razor blade, and then the preparation was inserted into the GHS-DPP imaging system.

Supplementary Table 1. The estimated number of ommatidia forming the DPP image.

**Microscope NA (in air)**	**N (wild-type ommatidia) (r = 2.8 µm)**			**N (*Spam* ommatidia) (r = 1.5 µm)**			**One wild-type rhabdomere (r = 0.78 µm)**		
	**contributing at least**								
	100%	50%	>0%	100%	50%	0%	100%	50%	>0%
0.001	0	7.3	15	0.11	3.2	6.2	0.54	1.8	3.1
0.01	0	8.0	16	0.21	3.7	7.1	0.65	2.2	3.8
0.05	0	11	23	0.67	6.3	12	1.3	4.4	7.4
0.1	0.47	17	33	1.9	11	20	4.4	9.1	14
0.2	4.1	32	61	11	26	41	17	25	32
0.4	38	90	143	56	84	112	68	83	97
0.6	115	195	274	146	189	231	165	187	209
0.8	268	380	492	314	374	434	341	372	403
0.95	521	671	821	585	665	745	622	663	705
