# Supplementary material for: High-speed imaging of light-induced photoreceptor microsaccades in compound eyes
Source: Commun Biol. 2022 Mar 3;5:203. doi: 10.1038/s42003-022-03142-0 (PMC8894348; doi:10.1038/s42003-022-03142-0)
Supplement: Supplementary file 3 — Description of Additional Supplementary Files [file 42003_2022_3142_MOESM3_ESM.pdf]

Description of Additional Supplementary Files

File name: Supplementary Video 1
Description: Step-by-step animated instructions for constructing a goniometric high speed deep pseudopupil (GHS-DPP) microscope system.

File name: Supplementary Video 2
Description: In vivo infrared imaging a Drosophila's photomechanical photoreceptor dynamics to UV-light flashes at different eye locations. The video shows how to integrate a map of microsaccade directions across the Drosophila eyes.

File name: Supplementary Video 3
Description: This animation shows how the developmentally rotated R1-R7/8 photoreceptor rhabdomere orientations at each eye position align with the frontally expanding optic flow. Note how the optic flow vectors at different eye locations cross the ommatidial R1-R7/8 rhabdomeres approximately perpendicular to their R1-R2-R3 axis.

File name: Supplementary Video 4
Description: Drosophila eye CG-model shows the deep pseudopupil principle and how the number of light-activated photoreceptors generating photomechanical microsaccades depends upon the local light stimulus's angular size.

File name: Supplementary Video 5
Description: GHS-DPP imaging photomechanical photoreceptor dynamics of five wild type and spam mutant Drosophila at different eye locations. The later animation section shows the comparison of their mean microsaccade direction maps.

File name: Supplementary Video 6
Description: This animation shows how the R1-R7/8 photoreceptor microsaccade directions and their developmentally rotated rhabdomere orientations align at each eye position, given as angular difference. Note how the microsaccades at different eye locations move along the 996 R1-R2-R3 axis.
