# Supplementary material for: High-speed imaging of light-induced photoreceptor microsaccades in compound eyes
Source: Commun Biol. 2022 Mar 3;5:203. doi: 10.1038/s42003-022-03142-0 (PMC8894348; doi:10.1038/s42003-022-03142-0)
Supplement: Supplementary file 10 — Reporting Summary [file 42003_2022_3142_MOESM10_ESM.pdf]

nature research

Corresponding author(s): Mikko Juusola

Last updated by author(s): Feb 3, 2022

Reporting Summary

Nature Research wishes to improve the reproducibility of the work that we publish. This form provides structure for consistency and transparency in reporting. For further information on Nature Research policies, see our Editorial Policies and the Editorial Policy Checklist.

Statistics

For all statistical analyses, confirm that the following items are present in the figure legend, table legend, main text, or Methods section.

n/a	Confirmed	
☐	☒	The exact sample size (n) for each experimental group/condition, given as a discrete number and unit of measurement
☐	☒	A statement on whether measurements were taken from distinct samples or whether the same sample was measured repeatedly
☐	☒	The statistical test(s) used AND whether they are one- or two-sided<br>*Only common tests should be described solely by name; describe more complex techniques in the Methods section.*
☒	☐	A description of all covariates tested
☐	☒	A description of any assumptions or corrections, such as tests of normality and adjustment for multiple comparisons
☐	☒	A full description of the statistical parameters including central tendency (e.g. means) or other basic estimates (e.g. regression coefficient) AND variation (e.g. standard deviation) or associated estimates of uncertainty (e.g. confidence intervals)
☐	☒	For null hypothesis testing, the test statistic (e.g. F, t, r) with confidence intervals, effect sizes, degrees of freedom and P value noted<br>*Give P values as exact values whenever suitable.*
☒	☐	For Bayesian analysis, information on the choice of priors and Markov chain Monte Carlo settings
☒	☐	For hierarchical and complex designs, identification of the appropriate level for tests and full reporting of outcomes
☐	☒	Estimates of effect sizes (e.g. Cohen's d, Pearson's r), indicating how they were calculated

Our web collection on statistics for biologists contains articles on many of the points above.

Software and code

Policy information about availability of computer code

Data collection	To enable non-specialist users to operate the GHS-DPP microsaccade imaging experiments, we created a free and open (GPLv3) recording software called Gonio Imsoft. Gonio Imsoft interfaced with the open-source microscopy software MicroManager to control the high-speed camera. In addition, it used the NI-DAQmx module (controlling the data acquisition) and the PySerial module (communicating with the Arduino microcontroller), reading out the rotary encoders. Gonio Imsoft ran on a Windows 10 platform. All code are available from hhttps://github.com/JuusolaLab/GHS-DPP_paper.
Data analysis	To quantify the 3-dimensional rhabdomere movement fields, we created a free and open (GPLv3) data analysis software called Gonio Analysis. It allows drawing regions of interest (ROIs) around the deep pseudopupil, performing motion analysis, and finally, translating the motion results from the camera image coordinates to the fly's 3D frame-of-reference, using the digitally read rotation stage values. Here, we shortly describe its data analysis principles. All code are available from hhttps://github.com/JuusolaLab/GHS-DPP_paper.<br>For making figures were used Origin software.

For manuscripts utilizing custom algorithms or software that are central to the research but not yet described in published literature, software must be made available to editors and reviewers. We strongly encourage code deposition in a community repository (e.g. GitHub). See the Nature Research guidelines for submitting code & software for further information.

Data

Policy information about availability of data

All manuscripts must include a data availability statement. This statement should provide the following information, where applicable:

- Accession codes, unique identifiers, or web links for publicly available datasets
- A list of figures that have associated raw data
- A description of any restrictions on data availability

https://github.com/JuusolaLab/Hyperacute_Stereopsis_paper

Field-specific reporting

Please select the one below that is the best fit for your research. If you are not sure, read the appropriate sections before making your selection.

☒ Life sciences ☐ Behavioural & social sciences ☐ Ecological, evolutionary & environmental sciences

For a reference copy of the document with all sections, see nature.com/documents/nr-reporting-summary-flat.pdf

Life sciences study design

All studies must disclose on these points even when the disclosure is negative.

Sample size	We mapped the compound eyes' microsaccade directions across 5 individual flies of each tested genotype.<br>The microsaccade dynamics were locally recorded from many hundreds of eye positions. At each locations, the recording were repeated 25 times.
Data exclusions	All completed experiments were included in this study.
Replication	Many experiments were performed separately by different individuals and their results were pooled.
Randomization	The order of testing different visual patterns in Drosophila learning experiments were randomised.
Blinding	N/A

Reporting for specific materials, systems and methods

We require information from authors about some types of materials, experimental systems and methods used in many studies. Here, indicate whether each material, system or method listed is relevant to your study. If you are not sure if a list item applies to your research, read the appropriate section before selecting a response.

Materials & experimental systems

n/a	Involved in the study	
☒	☐	Antibodies
☒	☐	Eukaryotic cell lines
☒	☐	Palaeontology and archaeology
☐	☒	Animals and other organisms
☒	☐	Human research participants
☒	☐	Clinical data
☒	☐	Dual use research of concern

Methods

n/a	Involved in the study	
☒	☐	ChIP-seq
☒	☐	Flow cytometry
☒	☐	MRI-based neuroimaging

Animals and other organisms

Policy information about studies involving animals; ARRIVE guidelines recommended for reporting animal research

Laboratory animals	We used wild-type and spam mutant Drosophila genotypes, obtained from standard laboratory stocks.
Wild animals	N/A
Field-collected samples	N/A
Ethics oversight	N/A

Note that full information on the approval of the study protocol must also be provided in the manuscript.
